# Phenotypes and Virulence among *Staphylococcus aureus* USA100, USA200, USA300, USA400, and USA600 Clonal Lineages

**DOI:** 10.1128/mSphere.00071-16

**Published:** 2016-06-08

**Authors:** Jessica M. King, Katarina Kulhankova, Christopher S. Stach, Bao G. Vu, Wilmara Salgado-Pabón

**Affiliations:** Department of Microbiology, University of Iowa, Carver College of Medicine, Iowa City, Iowa, USA; Providence Portland Medical Center

**Keywords:** *Staphylococcus aureus*, infective endocarditis, rabbit model, sepsis, superantigens, virulence factors

## Abstract

*Staphylococcus aureus* diseases affect ~500,000 individuals per year in the United States. Worldwide, the USA100, USA200, USA400, and USA600 lineages cause many of the life-threatening *S. aureus* infections, such as bacteremia, infective endocarditis, pneumonia, toxic shock syndrome, and surgical site infections. However, the virulence mechanisms associated with these clonal lineages, in particular the USA100 and USA600 isolates, have been severely understudied. We investigated the virulence of these strains, in addition to strains in the USA200, USA300, and USA400 types, in well-established *in vitro* assays and *in vivo* in the rabbit model of infective endocarditis and sepsis. We show in the infective endocarditis and sepsis model that strains in the USA100 and USA600 lineages cause high lethality and are proficient in causing native valve infective endocarditis. Strains with high cytolytic activity or producing toxic shock syndrome toxin 1 (TSST-1) or staphylococcal enterotoxin C (SEC) caused lethal sepsis, even with low cytolytic activity. Strains in the USA100, USA200, USA400, and USA600 lineages consistently contained genes that encode for the enterotoxin gene cluster proteins, SEC, or TSST-1 and were proficient at causing infective endocarditis, while the USA300 strains lacked these toxins and were deficient in promoting vegetation growth. The USA100, USA200, and USA400 strains in our collection formed strong biofilms *in vitro*, whereas the USA200 and USA600 strains exhibited increased blood survival. Hence, infective endocarditis and lethal sepsis are multifactorial and not intrinsic to any one individual clonal group, further highlighting the importance of expanding our knowledge of *S. aureus* pathogenesis to clonal lineages causative of invasive disease.

**IMPORTANCE** *S. aureus* is the leading cause of infective endocarditis in the developed world, affecting ~40,000 individuals each year in the United States, and the second leading cause of bacteremia (D. R. Murdoch et al., Arch Intern Med 169:463–473, 2009, http://dx.doi.org/10.1001/archinternmed.2008.603, and H. Wisplinghoff et al., Clin Infect Dis 39:309–317, 2004, http://dx.doi.org/10.1086/421946). Even with current medical advances, *S. aureus* bloodstream infections and infective endocarditis carry mortality rates of 20 to 66% (S. Y. Tong et al., Clin Microbiol Rev 28:603–661, 2015, http://dx.doi.org/10.1128/CMR.00134-14). *S. aureus* lineages associated with human disease worldwide include clonal complex 5 (CC5)/USA100, CC30/USA200, CC8/USA300, CC1/USA400, and CC45/USA600. The CC5/USA100, CC30/USA200, and CC45/USA600 lineages cause invasive disease yet remain poorly characterized. USA300 and cytotoxins are central to most *S. aureus* virulence studies, and yet, we find evidence that clonal groups are quite heterogeneous in parameters canonically used to measure virulence, including cytotoxicity, biofilm formation, and blood survival, and that the superantigen profile is an important parameter to consider when defining the virulence of *S. aureus* strains.

## Abstract (precis)

*S. aureus* is the leading cause of infective endocarditis in the developed world, affecting ~40,000 individuals each year in the United States, and the second leading cause of bacteremia (D. R. Murdoch et al., Arch Intern Med 169:463–473, 2009, http://dx.doi.org/10.1001/archinternmed.2008.603, and H. Wisplinghoff et al., Clin Infect Dis 39:309–317, 2004, http://dx.doi.org/10.1086/421946). Even with current medical advances, *S. aureus* bloodstream infections and infective endocarditis carry mortality rates of 20 to 66% (S. Y. Tong et al., Clin Microbiol Rev 28:603–661, 2015, http://dx.doi.org/10.1128/CMR.00134-14). *S. aureus* lineages associated with human disease worldwide include clonal complex 5 (CC5)/USA100, CC30/USA200, CC8/USA300, CC1/USA400, and CC45/USA600. The CC5/USA100, CC30/USA200, and CC45/USA600 lineages cause invasive disease yet remain poorly characterized. USA300 and cytotoxins are central to most *S. aureus* virulence studies, and yet, we find evidence that clonal groups are quite heterogeneous in parameters canonically used to measure virulence, including cytotoxicity, biofilm formation, and blood survival, and that the superantigen profile is an important parameter to consider when defining the virulence of *S. aureus* strains.

## INTRODUCTION

*Staphylococcus aureus* is a Gram-positive opportunistic pathogen that colonizes the mucosal surfaces and skin of approximately 30% of humans (1). *S. aureus* causes life-threatening infections worldwide, such as necrotizing and hemorrhagic pneumonias, osteomyelitis, menstrual toxic shock syndrome (TSS), TSS as a consequence of any infection, bloodstream infections, and infective endocarditis (2). The emergence of multidrug resistance has made *S. aureus* infections increasingly difficult to treat, especially those acquired in health care settings. In the community, methicillin-resistant *S. aureus* (MRSA) contributes significantly to the burden of *S. aureus* invasive disease (3).

*S. aureus* lineages circulating worldwide and frequently associated with serious illness include clonal complex 5 (CC5)/USA100, CC30/USA200, CC8/USA300, CC1/USA400, and CC45/USA600 (4, 5). *S. aureus* USA100 clones are the predominant lineage colonizing the human nares in the United States and are widespread in hospitals but also present in the community (6, 7). Importantly, USA100 strains are a leading cause of invasive disease among MRSA isolates and represent the majority of vancomycin-resistant/-intermediate *S. aureus* isolates (8, 9). In northern Europe, USA600 strains are the predominant human colonizers and were epidemic throughout Germany and the Netherlands (10). Recent reports noted increased detection and causation of lethal invasive disease by strains of this lineage in the United States (10–12). Another clonal group with widespread presence in health care settings and the community is the USA200 lineage. These strains colonize mucosal surfaces and cause life-threatening infections, most notably menstrual TSS, which occurs in otherwise healthy women (13). USA400 strains are also community associated, are frequently isolated in the upper Midwest and southwestern Alaska, and have emerged as the predominant MRSA type in western Canada (14, 15).

USA300 is the current community epidemic lineage in the United States, predominantly causing severe skin and soft tissue infections (2). The USA300 lineage is the most well-studied clonal group, and strains from this group often produce high levels of cytotoxins, such as alpha-toxin and Panton-Valentine leukocidin (2). Perhaps as a consequence, past studies have mostly implicated cytotoxins in *S. aureus* virulence. However, cytotoxic profiles of *S. aureus* strains are often quite heterogeneous, varying remarkably even within each clonal group, and there is new evidence for an inverse correlation between *S. aureus* toxicity and virulence (16). Studies showed that low-toxicity isolates had a higher propensity to cause invasive infections like bacteremia, and patient mortality was four times higher in patients with nosocomial pneumonia caused by *S. aureus* isolates with low cytotoxic activity.

Strikingly, while USA100/CC5, USA200/CC30, and USA600/CC45 lineages cause the majority of bloodstream infections and infective endocarditis worldwide, these strains remain largely uncharacterized (4, 5, 17–20). Furthermore, strains in the USA200 and USA400 clonal groups are commonly defined as strains with reduced virulence, as tested in rodent models (21, 22). However, these strains cause lethal disease in healthy humans, such as necrotizing/hemorrhagic pneumonias with lethal TSS (23) and menstrual TSS (24). Conflicting outcomes obscure the identification of *S. aureus* virulence factors that lead to disease and increase mortality.

Various studies have linked specific *S. aureus* clonal groups with particular infections or patient outcomes, while others have found a causal association of specific virulence factors with disease (25). *S. aureus* has a myriad of virulence factors used to silently colonize, invade, survive in the bloodstream, and establish an infection niche—many of which are redundant in function and variably encoded and produced (26). Hence, the combination of virulence factors conferring specific physiologic functions is more likely to define *S. aureus* ability to cause disease and its severity (besides host-specific risk factors). This combination may be linked to a particular clonal group or be common to several lineages.

To elucidate characteristics common to invasive *S. aureus* strains, we characterized a subset of isolates from five United States types associated with human disease, many of which (such as USA100 and USA600) are clinically important yet inadequately characterized (Table 1). USA100 and USA600 clinical isolates were compared to strains in the USA200, USA300 and USA400 clonal groups. We determined cytolytic potential, biofilm formation, and blood survival of each isolate, as well as their virulence in a rabbit model of infective endocarditis and sepsis.

**TABLE 1:** *S.* aureus clinical isolates

Characteristic*^a^*	No. of isolates (%)
PFGE type	
USA100	12 (25)
USA200	8 (16)
USA300	12 (25)
USA400	6 (12)
USA600	9 (18)
Nontypeable	2 (4)
Methicillin status	
MRSA	39 (80)
MSSA	10 (20)
Isolation site	
Blood	9 (18)
Lungs	12 (25)
Wound/abscess/tissue	13 (27)
Vagina/mTSS	5 (10)
Nares	2 (4)
Other sterile site	8 (16)
Total	49 (100)

a PFGE, pulsed-field gel electrophoresis; MSSA, methicillin-susceptible *S. aureus*; mTSS, menstrual toxic shock syndrome.

## RESULTS

### United States clonal groups that cause invasive disease exhibit specific superantigen gene profiles.

**FIG 1:**
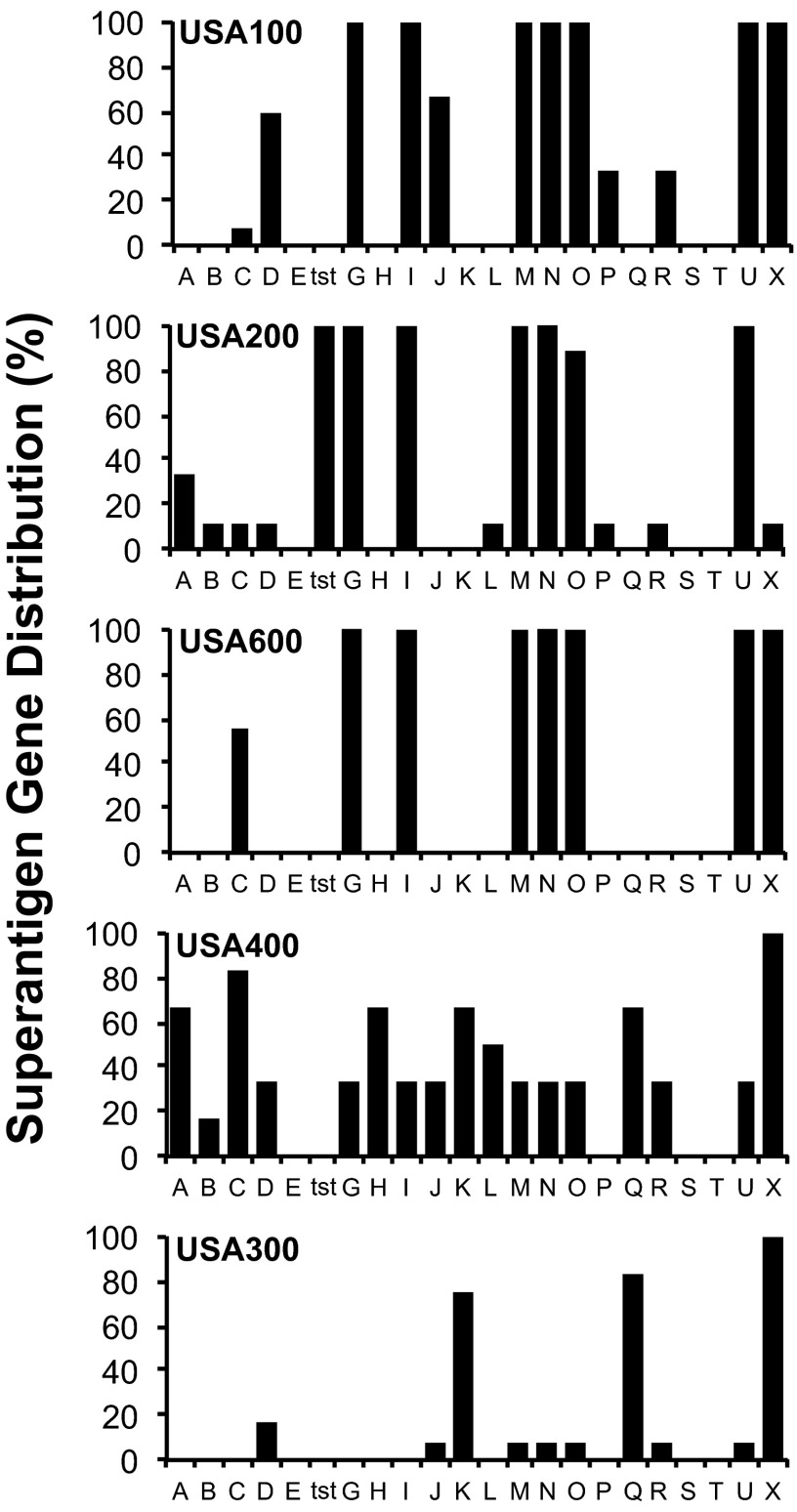
Superantigen (SAg) gene profiles among *S. aureus* clinical isolates from multiple clonal groups. A total of 49 strains were screened for the presence of 22 SAg genes by PCR amplification with gene-specific primers.

There is a strong association between select superantigens (SAgs) and invasive *S. aureus* diseases like infective endocarditis, pneumonia, and both menstrual- and nonmenstrual toxic shock syndrome (4, 6, 27). Therefore, we used PCR to screen strains for the presence of 22 individual SAg genes (Fig. 1; Table 2). We detected staphylococcal enterotoxin (SE) G (SEG), SEI, staphylococcal enterotoxin-like M (SE*l*-M), SE*l*-N, SE*l*-O, and SE*l*-U genes, collectively known as the enterotoxin gene cluster (*egc*), in 100% of isolates from the USA100, USA200, and USA600 lineages. All USA200 strains encoded toxic shock syndrome toxin 1 (TSST-1), their hallmark SAg. SEC was not only highly prevalent in USA400 isolates but was also present in ~60% of USA600 strains and in ~10% of USA100 and USA200 strains. USA300 strains encoded the least number of SAgs and lacked those frequently associated with invasive disease. The majority of USA300 strains encoded SE*l-*K, SE*l-*Q, and SE*l*-X. These results demonstrate the high frequency of SAg genes in *S. aureus* backgrounds associated with invasive and life-threatening diseases.

**TABLE 2:** *Staphylococcus aureus* strains tested and their corresponding enterotoxin gene profiles

Clonal group	*S. aureus* strain	Methicillin status*^a^*	Source or origin*^b^*	Superantigen gene profile
USA100	IA116	MRSA	Blood	*sed*, *seg*, *sei*, *selj*, *selm*, *seln*, *selo*, *selu*, *selx*
	IA132	MRSA	Blood	*seg*, *sei*, *selm*, *seln*, *selo*, *selu*, *selx*
	IA197	MRSA	Blood	*sed*, *seg*, *sei*, *selj*, *selm*, *seln*, *selo*, *selu*, *selx*
	IA1624	MRSA	Blood	*sed*, *seg*, *sei*, *selj*, *selm*, *seln*, *selo*, *selu*, *selx*
	IA140	MRSA	Lower respiratory tract	*seg*, *sei*, *selm*, *seln*, *selo*, *selp*, *selu*, *selx*
	IA1061	MRSA	Lower respiratory tract	*seg*, *sei*, *selm*, *seln*, *selo*, *selp*, *selu*, *selx*
	IA1495	MRSA	Lower respiratory tract	*sed*, *seg*, *sei*, *selj*, *selm*, *seln*, *selo*, *selu*, *selx*
	IA2636	MRSA	Lower respiratory tract	*seg*, *sei*, *selm*, *seln*, *selo*, *selu*, *selx*
	IA202	MRSA	Other sterile site	*sec*, *seg*, *sei*, *selm*, *seln*, *selo*, *selu*, *selx*
	IA209	MRSA	Other sterile site	*sed*, *selr*, *seg*, *sei*, *selj*, *selm*, *seln*, *selo*, *selp*, *selu*, *selx*
	IA217	MRSA	Other sterile site	*sed*, *seg*, *sei*, *selj*, *selm*, *seln*, *selo*, *selu*, *selx*
	IA2024	MRSA	Other sterile site	*sed*, *seg*, *sei*, *selj*, *selm*, *seln*, *selo*, *selu*, *selx*
USA200	IA1101	MSSA	Blood	*tstH*, *sea*, *seg*, *sei*, *selm*, *seln*, *selo*, *selu*
	MNPE	MSSA	Pneumonia/lethal TSS	*tstH*, *sec*, *seg*, *sei*, *selm*, *seln*, *selo*, *selu*, *sell*
	MN11	MSSA	Vaginal/mTSS	*tstH*, *sed*, *seg*, *sei*, *selm*, *seln*, *selo*, *selu*, *selx*
	CDC587	MSSA	Vaginal/mTSS	*tstH*, *seg*, *sei*, *selm*, *seln*, *selo*, *selu*
	MNWH	MRSA	Vaginal/mTSS	*tstH*, *sea*, *seg*, *sei*, *selm*, *seln*, *selo*, *selu*
	MNPA	MRSA	Vaginal/mTSS	*tstH*, *seg*, *sei*, *selm*, *seln*, *selo*, *selu*
	MN8	MSSA	Vaginal/mTSS	*tstH*, *sea*, *seg*, *sei*, *selm*, *seln*, *selo*, *selu*
	IA2624	MRSA	Other sterile site	*tstH*, *seg*, *sei*, *selm*, *seln*, *selu*
USA300	IA2980	MRSA	Blood	*selk*, *selq*, *selx*
	IA1643	MSSA	Blood	*selx*
	IA2995	MRSA	Blood	*sed*, *selj*, *selk*, *selq*, *selr*, *selx*
	PSLV	MSSA	Lethal pneumonia	*selx*
	PSMG	MSSA	Pneumonia	*selm*, *seln*, *selo*, *selu*, *selx*
	IA1012	MRSA	Wound/abscess	*selk*, *selq*, *selx*
	IA364	MRSA	Wound/abscess	*selk*, *selq*, *selx*
	IA3048	MRSA	Wound/abscess	*selk*, *selq*, *selx*
	LAC, Fitz	MRSA	Skin infection	*selk*, *selq*, *selx*
	IA2816	MRSA	Infected tissue	*sed*, *selk*, *selq*, *selx*
	IA2030	MRSA	Infected tissue	*selk*, *selq*, *selx*
	IA65	MRSA	Other sterile site	*selk*, *selq*, *selx*
USA400	MW2	MRSA	Pneumonia/lethal TSS	*sea*, *sec*, *selh*, *selk*, *sell*, *selq*, *selx*
	PSAG	MRSA	Pneumonia/lethal TSS	*sec*, *sed*, *seg*, *sei*, *selj*, *selm*, *seln*, *selo*, *selr*, *selu*, *selx*
	PSKN	MRSA	Pneumonia/lethal TSS	*sea*, *sec*, *selh*, *selk*, *sell*, *selq*, *selx*
	PSGN	MRSA	Pneumonia/TSS	*sea*, *sec*, *selh*, *selk*, *sell*, *selq*, *selx*
	C99-529	MRSA	Pneumonia/lethal TSS	*sea*, *seb*, *selh*, *selk*, *selq*, *selx*
	IA96	MSSA	Wound/abscess	*sec*, *sed*, *seg*, *sei*, *selj*, *selm*, *seln*, *selo*, *selu*, *selx*
USA600	IA2479	MRSA	Blood	*sec*, *seg*, *sei*, *selm*, *seln*, *selo*, *selu*, *selx*
	IA1234	MRSA	Wound/abscess	*sec*, *seg*, *sei*, *selm*, *seln*, *selo*, *selu*, *selx*
	IA746	MRSA	Wound/abscess	*sec*, *seg*, *sei*, *selm*, *seln*, *selo*, *selu*, *selx*
	IA2341	MRSA	Wound/abscess	*sec*, *seg*, *sei*, *selm*, *seln*, *selo*, *selu*, *selx*
	IA1871	MRSA	Wound/abscess	*seg*, *sei*, *selm*, *seln*, *selo*, *selu*, *selx*
	IA36	MRSA	Wound/abscess	*seg*, *sei*, *selm*, *seln*, *selo*, *selu*, *selx*
	IA1471	MRSA	Infected tissue	*sec*, *seg*, *sei*, *selm*, *seln*, *selo*, *selu*, *selx*
	IA87	MRSA	Nasal	*seg*, *sei*, *selm*, *seln*, *selo*, *selu*, *selx*
	IA88	MRSA	Nasal	*seg*, *sei*, *selm*, *seln*, *selo*, *selu*, *selx*
Historical	Col	MRSA	Operating theater, London, UK, 1960s	*seb*, *selk*, *selq*, *selx*
	RN450	MSSA	Corneal ulcer, NCTC 8325 variant, Oxford, UK, 1943	*selx*

a MSSA, methicillin-susceptible *S. aureus*.

b mTSS, menstrual toxic shock syndrome.

Furthermore, we measured the production levels of TSST-1, SEB, SEC, and SE*l*-X in the *S. aureus* isolates used for *in vivo* studies and found the strains to produce 5 to 50 µg/ml TSST-1, 50 µg/ml SEB, 58 to 81 µg/ml SEC, and 70 to 180 ng/ml SE*l*-X. The production of TSST-1, SEB, and SEC in liquid culture has been quantified previously in *S. aureus* isolates from atopic dermatitis, vaginal mucosa, and diabetic foot ulcers, where strains produced 3 to 39 µg/ml TSST-1, 25 to 120 µg/ml SEB, and 10 to 120 µg/ml SEC (28). SE*l*-X was also quantified in isolates from diabetic foot infections, with production ranging from 10 to 20 µg/ml. Therefore, the levels of SAg production in our strain collection are consistent with those previously reported. However, compared to diabetic foot infection isolates, our tested isolates produced much lower levels of SE*l*-X.

### *S. aureus* strains exhibit differential hemolysis of erythrocytes.

**FIG 2:**
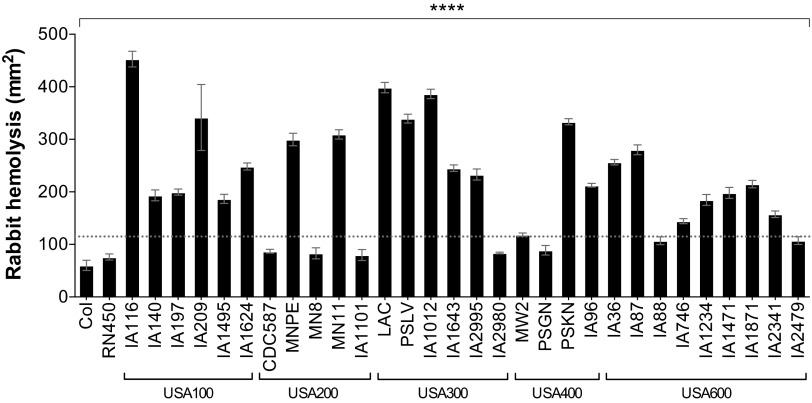
*S. aureus* clinical isolates exhibit differential hemolysis of rabbit erythrocytes within clonal lineages. Levels of alpha-toxin production as measured in a rabbit erythrocyte lysis assay are shown. Overnight cultures of *S. aureus* were washed and spotted onto tryptic soy agar plates containing 5% rabbit blood. Zones of hemolysis were determined after overnight growth. Data are presented as mean values ± standard errors of the means. Values above the dotted line are significantly different than those below. ****, *P* < 0.0001 by one-way ANOVA and Holm-Sidak multiple-comparison test; *P* values of ≤0.05 are considered statistically significant.

Alpha-toxin is an important *S. aureus* exotoxin that is commonly used to assess strain toxicity and virulence potential (26, 29). Alpha-toxin is a homoheptameric, pore-forming cytolysin that is variably produced and exhibits toxicity to various host cells, including immune cells (29, 30). We tested the hemolytic potential of *S. aureus* strains in an *in vitro* rabbit erythrocyte lysis assay, where *S. aureus* cultures are spotted onto 5% rabbit blood plates and lytic zones measured after overnight incubation. Rabbit erythrocytes are very sensitive to alpha-toxin but more resistant to other *S. aureus* hemolysins, such as beta-toxin (31, 32). The USA100 and USA300 strains in our collection produced the largest zones of hemolysis on rabbit blood plates, averaging 270 ± 44 mm^2^ (mean ± standard deviation) and 244 ± 45 mm^2^, respectively, suggesting high alpha-toxin production (Fig. 2). Overall, 83% of USA300 strains had hemolysis zones higher than 200 mm^2^. While variability was seen among strains in each clonal group, the USA200 and USA400 isolates in our collection showed the most variability (Fig. 2). Strains in these two lineages exhibited either high hemolysis (above 200 mm^2^) or very low hemolysis (below 100 mm^2^). Strains were also screened for the presence of *hla*, *hlb*, and *pvl* (Table 3). All strains tested positive for the presence of *hla* and *hlb*. Of the USA300 strains, 5/6 were *pvl* positive, and of the USA400 strains, 3/4 strains were positive for *pvl.* None of the USA100, USA200, or USA600 strains in our collection contained *pvl*.

**TABLE 3:** *Staphylococcus aureus* strains tested and their corresponding cytolysin gene profiles

Clonal group	*S. aureus* strain	Gene profile
USA100	IA116	*hla*, *hlb*^a^
	IA140	*hla*, *hlb*^a^
	IA197	*hla*, *hlb*^a^
	IA209	*hla*, *hlb*^a^
	IA1495	*hla*, *hlb*^a^
	IA1624	*hla*, *hlb*^a^
USA200	CDC587	*hla*, *hlb*^a^
	MNPE	*hla*, *hlb*^a^
	MN8	*hla*, *hlb*^a^
	MN11	*hla*, *hlb*^a^
	IA1101	*hla*, *hlb*^a^
USA300	LAC	*hla*, *hlb*^a^, *pvl*
	PSLV	*hla*, *hlb*^a^, *pvl*
	IA1012	*hla*, *hlb*^a^, *pvl*
	IA1643	*hla*, *hlb*^a^
	IA2995	*hla*, *hlb*^a^, *pvl*
	IA2980	*hla*, *hlb*^a^, *pvl*
USA400	MW2	*hla*, *hlb*^a^, *pvl*
	PSGN	*hla*, *hlb*^a^, *pvl*
	PSKN	*hla*, *hlb*^a^, *pvl*
	IA96	*hla*, *hlb*^a^
USA600	IA36	*hla*, *hlb*
	IA87	*hla*, *hlb*
	IA88	*hla*, *hlb*
	IA746	*hla*, *hlb*
	IA1234	*hla*, *hlb*
	IA1471	*hla*, *hlb*
	IA1871	*hla*, *hlb*
	IA2341	*hla*, *hlb*
	IA2479	*hla*, *hlb*
Historical	Col	*hla*, *hlb*
	RN450	*hla*, *hlb*^a^

a Previously documented (52).

### Biofilm formation is variable among *S. aureus* strains from different clonal groups.

**FIG 3:**
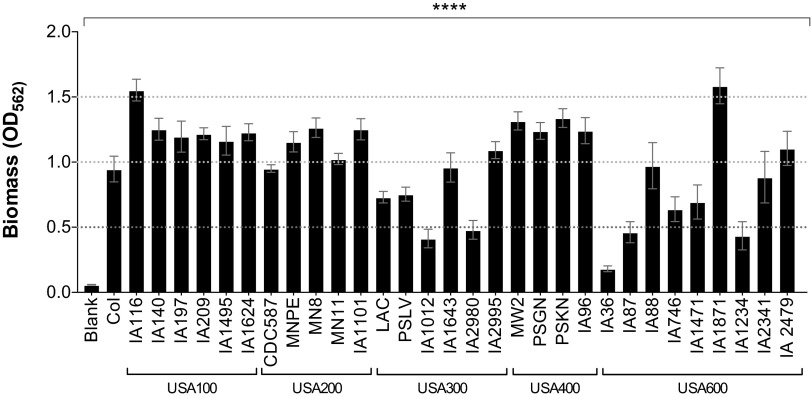
Strains within the USA300 and USA600 clonal groups form the weakest biofilms. Levels of biofilm formation on plasma-coated microtiter plates are shown. Data are presented as mean values ± standard errors of the means. Values separated by dotted lines are significantly different. ****, *P* < 0.0001 by one-way ANOVA and Holm-Sidak multiple-comparison test; *P* values of ≤0.05 are considered statistically significant.

Biofilm formation is an important component of many bacterial infections. The ability of a bacterial strain to form a biofilm is often one of the parameters by which its virulence is assessed. Therefore, we tested strains from different United States types in a well-established biofilm assay with plasma-coated microtiter plates. Strains within the USA100, USA200, and USA400 clonal lineages in our collection formed the strongest biofilms and exhibited the lowest intraclonal variability (Fig. 3). USA300 and USA600 strains had the largest intraclonal variability, and those strains that formed the weakest biofilms belong to these two clonal groups (Fig. 3). However, the USA600 lineage also had strains that produced some of the strongest biofilms.

### USA200 and USA600 strains demonstrate the highest overall survival in rabbit blood.

**FIG 4:**
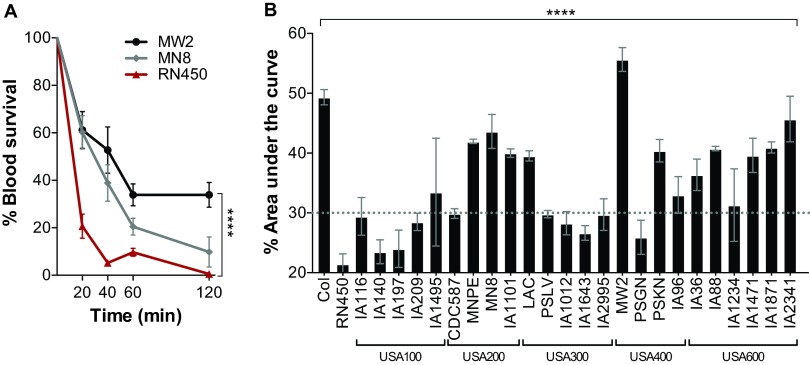
Strains in the USA200 and USA600 lineages exhibit the highest survival in blood. Freshly collected heparinized blood was inoculated with the indicated *S. aureus* strains at concentrations of 1 × 10^7^ to 5 × 10^7^ CFU/ml and incubated for a total of 120 min. *S. aureus* viability was determined at 0, 20, 40, 60, and 120 min. (A) Representative isolates with significantly different rates of survival in blood. Data are plotted as the percentages of viable bacteria recovered from the initial inoculum. (B) Blood survival of *S. aureus* isolates from multiple clonal groups. The area under each survival curve was calculated using GraphPad Prism software and plotted as the average survival for each strain. Data are plotted as the percentage of the area under the curve for each isolate. Values above the dotted line are significantly different than those below. Data are presented as mean values ± standard errors of the means. ****, *P* < 0.0001 by one-way ANOVA and Holm-Sidak multiple-comparison test; *P* values of ≤0.05 are considered statistically significant.

Survival in the blood is critical for *S. aureus* dissemination, hematogenous colonization, and persistent bacteremia. It also reflects the production of a myriad of virulence factors that act to resist host immune defenses either by direct mechanisms or indirectly as a result of bacterial clumping. To address this, we tested the ability of *S. aureus* to survive in rabbit blood in an *in vitro* assay (Fig. 4). We chose to use rabbit blood rather than human blood to more directly correlate with experiments performed in the rabbit model of infective endocarditis and sepsis.

Freshly collected heparinized rabbit blood was inoculated with approximately 2.5 × 10^7^ CFU. Samples were collected at 0, 20, 40, 60, and 120 min, and percent survival over time calculated and plotted to generate a survival curve as represented in Fig. 4A. Strain RN450 demonstrated the weakest survival, showing survival below 20% only 20 min after inoculation (Fig. 4A). The area under each survival curve was calculated and plotted as the average survival for each strain (Fig. 4B). Strains Col and MNPE (USA200), MN8 (USA200), IA1101 (USA200), LAC (USA300), MW2 (USA400), IA1471 (USA600), IA1871 (USA600), and IA2341 (USA600) were exceptional at surviving in blood, averaging higher than 40% survival over the 2-h period (Fig. 4B). Overall, strains in the USA200 and USA600 lineages in our collection exhibited the highest survival in blood, with 75% and 100% of strains, respectively, exhibiting greater than 30% survival on average over the 2-h period (Fig. 4B). The USA100 lineage exhibited the overall lowest survival in blood, with 80% of the strains exhibiting less than approximately 30% survival. Intraclonal variability was seen among strains in nearly all lineages, where at least one strain demonstrated significantly higher or lower survival than the rest of the strains in the clonal group (i.e., percent survival fell on the opposite side of the dotted line) (Fig. 4B).

### *S. aureus* USA100 and USA600 cause infective endocarditis.

**FIG 5:**
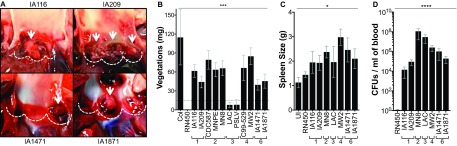
The ability of USA100 and USA600 strains to cause infective endocarditis is similar to that of well-established USA200 and USA400 strains in the rabbit model of infective endocarditis and sepsis. (A) Representative images of aortic vegetations caused by USA100 strains IA116 and IA209 (top) and USA600 strains IA1471 and IA1871 (bottom). Dotted lines outline valve cusps. Arrows indicate vegetations. (B) Total mean weights of vegetations dissected from aortic valves (±standard errors of the means) after intravenous inoculation with 1 × 10^7^ to 5 × 10^8^ CFU, as described in Materials and Methods. Values above the dotted line are significantly different than those below. ***, *P* = 0.0002 by one-way ANOVA and Holm-Sidak multiple-comparison test. (C) Enlargement of the spleen resulting from *S. aureus* infection. UI, uninfected. *, *P* = 0.01 by one-way ANOVA. (D) Bacterial counts per milliliter of blood recovered from rabbits postmortem. ****, *P* < 0.0001 by one-way ANOVA; *P* values of ≤0.05 are considered statistically significant. 1, USA100; 2, USA200; 3, USA300; 4, USA400; 6, USA600.

*S. aureus* USA200 and USA400 strains have previously been shown to be excellent at causing infective endocarditis, while *S. aureus* USA300 strains were deficient or formed very small vegetations (33). *S. aureus* vegetations are composed of aggregates of fibrin, platelets, erythrocytes, and bacteria on heart valves and are pathognomic of infective endocarditis. Some of the most common infective endocarditis isolates belong to the CC5/USA100 and CC45/USA600 lineages but are yet to be tested in experimental native valve infective endocarditis. We took a subset of strains in our collection demonstrating different origins of isolation, SAg profiles, and hemolysis levels (Table 4) and tested them in the rabbit infective endocarditis and sepsis model (Fig. 5). Consistent with epidemiological studies, rabbits infected with USA100 and USA600 isolates had large vegetations on aortic valves with the classic appearance of fibrinous endocarditis lesions (Fig. 5A). Strains IA209 (USA100), IA1471 (USA600), and IA1871 (USA600) produced vegetations of similar sizes (approximately 45 mg), while IA116 (USA100) on average caused slightly larger vegetations (approximately 60 mg) (Fig. 5B). Most strains caused infective endocarditis in the 4-day experimental period when administered at 2 × 10^7^ to 5 × 10^7^ CFU total. Strains CDC587 (USA200), MN8 (USA200), and IA1871 (USA600) caused infective endocarditis at a dose of 2 × 10^8^ to 5 × 10^8^ CFU, and strain Col caused it at 5 × 10^8^ to 1 × 10^9^ CFU. Strain RN450 and the USA300 strains LAC and PSLV were severely deficient in infective endocarditis development regardless of the infection dose (Fig. 5B). Rabbits infected with strain LAC (USA300) developed significant splenomegaly (Fig. 5C), and bacteria were recovered in significant numbers from the bloodstream (Fig. 5D), similar to other strains that produced large vegetations on heart valves and consistent with blood survival studies (Fig. 4). These results suggest that bacteremia alone is not sufficient to promote the growth of vegetations on heart valves.

**TABLE 4:** Characteristics of strains tested in the infective endocarditis and sepsis model

USA type	Strains tested in IE and sepsis	Methicillin status*^a^*	Origin*^b^*	Amt(s) of superantigen(s) produced*^c^*	Rabbit hemolysis levels*^d^*
USA100	IA116	MRSA	Blood	NA	High
	IA209	MRSA	Other sterile site	NA	High
USA200	MN8	MSSA	Vaginal/mTSS	TSST-1 (20–50 µg/ml)	Low
	MNPE	MSSA	Post-influenza pneumonia/lethal TSS	TSST-1 (1–15 µg/ml), SEC (60–100 µg/ml)	High
	CDC587	MSSA	Vaginal/mTSS	TSST-1 (5 µg/ml)	Low
USA300	LAC	MRSA	Skin infection	SE*l*-X (180 ng/ml)	High
	PSLV*^e^*	MSSA	Necrotizing lethal pneumonia	SE*l*-X (180 ng/ml)	High
USA400	MW2	MRSA	Necrotizing pneumonia/lethal TSS	SEC (80–120 µg/ml), SE*l*-X (70–120 ng/ml)	Low
	C99-529	MRSA	Necrotizing pneumonia/lethal TSS	SEB (50 µg/ml)	NA
USA600	IA1471	MRSA	Infected tissue	SEC (30 µg/ml)	Medium
	IA1871	MRSA	Wound/abscess	NA	Medium

a MSSA, methicillin-susceptible *S. aureus*.

b mTSS, menstrual toxic shock syndrome.

c NA, not applicable.

d Comparison across all strains tested.

e Same as MN(Minnesota)Levy.

### Lethality due to *S. aureus* bacteremia is independent of United States clonal lineage.

**FIG 6:**
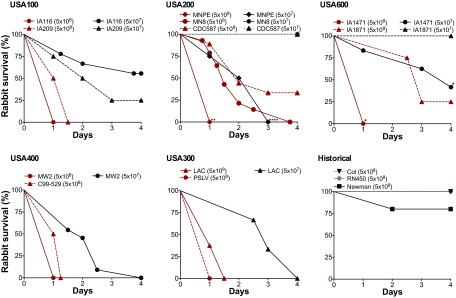
*S. aureus* strains from multiple clonal groups cause lethal sepsis in the infective endocarditis and sepsis model. Rabbits were infected intravenously with 5 × 10^7^ or 5 × 10^8^ CFU of the indicated strains, and percent survival was measured over 4 days. Asterisks indicate statistical significance of survival curves from rabbits infected with strains within the same clonal group and at the same dose (multiple curve comparison and log-rank Mantel-Cox test). **, *P* = 0.005 (USA200 strains at 5 × 10^8^ CFU); ****, *P* < 0.0001 (USA200 strains at 5 × 10^7^ CFU); *, *P* = 0.025 (USA600 strains at 5 × 10^8^ CFU); *, *P* = 0.036 (USA600 strains at 5 × 10^7^ CFU). The survival curves for strains MN8, CDC587, and IA1871 are significantly different than all other curves at the same infection dose (*P* ≤ 0.02 at 5 × 10^8^ CFU and *P* ≤ 0.04 at 5 × 10^7^ CFU). *P* values of ≤0.05 are considered statistically significant.

With the exception of *S. aureus* strain RN450, the strains tested in the infective endocarditis and sepsis model are proficient in persisting or surviving in the bloodstream, which besides infective endocarditis can lead to deep tissue toxicities, such as acute kidney injury and ischemic liver lesions, and result in significant mortality. In the infective endocarditis and sepsis model, intravenous administration of *S. aureus* strains at a dose of 5 × 10^8^ CFU induced high lethality in rabbits, where 8 of the 15 strains tested led to death in 24 to 36 h (Fig. 6). These strains represented all five United States clonal groups and included IA116 (USA100), IA209 (USA100), MNPE (USA200), IA1471 (USA600), MW2 (USA400), C99-529 (USA400), LAC (USA300), and PSLV (USA300). However, other strains were also highly lethal over the 4-day experimental period. Strains MN8 (USA200), CDC587 (USA200), and IA1871 (USA600) exhibited >60% lethality, with strain MN8 causing 100% lethality by day 4 (Fig. 6). In stark contrast, strain Newman exhibited <25% lethality, while strains RN450 and Col were not lethal (Fig. 6). A 1-log decrease in the infection dose (5 × 10^7^ CFU) still led to lethality in rabbits infected with *S. aureus* strains IA116 (30% lethality), IA209 (75% lethality), MNPE (100% lethality), IA1471 (60% lethality), MW2 (100% lethality), and LAC (100% lethality) (Fig. 6). Of importance, strains such as MN8 and MW2 caused significant lethality even though they exhibit decreased cytotoxicity, while strains such as LAC and PSLV exhibited significant lethality even though they are deficient in vegetation growth. Similarly, strains Col and Newman caused significant vegetation growth even though they induced no lethality or significantly reduced lethality at the infectious dose tested.

## DISCUSSION

*S. aureus* presents a significant clinical and public health problem, causing some of the most severe hospital- and community-associated illnesses and affecting approximately 500,000 individuals each year in the United States (34). *S. aureus* is the leading cause of infective endocarditis, accounting for about 40,000 cases per year (34–37), and is the second leading cause of sepsis (34, 38, 39). Contributing to these infections are both methicillin-susceptible and resistant strains of multiple *S. aureus* lineages, including the largely uncharacterized USA100 and USA600 strains. The USA100, USA200, and USA600 lineages also cause the majority of bloodstream infections and infective endocarditis worldwide (4, 18–20). Over the years, *S. aureus* virulence has been defined mostly by the cytotoxic capacity of any given strain (40). However, clinical data show that cytotoxicity is inversely correlated with strain invasiveness, where high cytolysin production is associated with decreased mortality (16, 20). Here, we characterized *S. aureus* strains from five United States types associated with human disease, expanding the characterization beyond cytotoxicity and the USA300 lineage to elucidate characteristics common to invasive *S. aureus* strains.

Currently, highly cytotoxic strains, such as USA300 strains, are considered hypervirulent, and low-cytotoxicity strains, such as USA200 strains, are considered less virulent (16, 21, 22). However, many strains from the USA200 clonal group produce cytotoxins at low levels yet cause lethal diseases in humans (4, 20). Some of the disconnect between low cytotoxicity and virulence of *S. aureus* is that many studies do not account for the production of SAgs, particularly TSST-1, SEB, or SEC, known to be causative agents of systemic inflammatory syndromes and toxic shock (41). In our studies, we provide evidence that strains with low to moderate levels of cytolytic activity, such as MN8 (USA200), MW2 (USA400), and IA1471 (USA600), exhibit high lethality in the rabbit infective endocarditis and sepsis model. Strains MN8, MW2, and IA1471 produce TSST-1 or SEC at high levels (above 20 µg/ml in liquid culture) (28, 42, 43). These results are consistent with the known virulence of MN8 and MW2 in humans, where MN8 was the causative agent of menstrual toxic shock syndrome and MW2 caused pneumonia with lethal toxic shock in a child (both without established risk factors) (23, 44). Previous studies have also demonstrated that in the context of vaginal colonization, pneumonia, or skin/soft tissue infections, the production of TSST-1, SEB, or SEC leads to systemic toxicity in humans and is associated with poor outcomes (16, 25, 45).

Conversely, there are also strains with high cytolytic activity that contain the enterotoxin gene cluster or encode SAgs that are produced at low levels (ng/ml in liquid culture), such as SE*l-*X, SE*l-*K, and SE*l-*Q, and also exhibit high lethality (42, 43). These include LAC (USA300), IA116 (USA100), and IA209 (USA100). We recognize that bacteremia/sepsis models do not account for strain invasiveness or ability to cause systemic disease from mucosal or tissue surfaces. The data suggest that if these strains are able to reach the systemic circulation, they can impose great damage to the host. Therefore, we speculate that the inverse correlation between high cytotoxicity and mortality observed in human infections is due to the localized and highly inflammatory effects of cytolysins (as observed in skin/soft tissue infections) in the absence of SAg production at higher levels.

Recent studies comparing the genotypes of *S. aureus* infective endocarditis isolates to those of skin/soft tissue infection isolates indicated a strong association between infective endocarditis and a select group of staphylococcal SAgs, where 18 to 25% of strains encoded SEC, 9 to 20% encoded TSST-1, and 58 to 90% contained the enterotoxin gene cluster (4, 5, 27). Consistent with epidemiological studies, the *S. aureus* USA100, USA200, USA400, and USA600 isolates contained the enterotoxin gene cluster or encoded SEC and/or TSST-1 and formed large vegetations in the rabbit model of infective endocarditis. These strains were isolated from various infection sites, including skin, lungs, vagina, and blood, and all were able to cause infective endocarditis. Isolates belonging to the USA300 clonal group encoded the lowest number of SAgs and lacked those associated with infective endocarditis, perhaps correlating with their poor ability to form vegetations on heart valves. We recently reported on the critical requirement of SEC for native valve infection and sequelae in infective endocarditis in rabbits (42), and others have reported on a similar requirement for TSST-1 (46). The underlying mechanism that defines an *S. aureus* strain’s potential for infective endocarditis development will be a focus of future studies. However, these results highlight that *S. aureus* isolates from clonal lineages that are severely understudied cause one of the most deadly *S. aureus* diseases and induce high lethality in an animal model that closely mimics human disease (2, 43).

To further characterize strains in the USA100 and USA600 lineage, we performed *in vitro* tests routinely used as parameters of virulence, as follows: blood hemolysis (as a measure of cytotoxic potential), biofilm formation (as a measure of tissue adherence/colonization), and blood survival (as a measure of ability to resist immune attack). Interestingly, we found clonal group heterogeneity in each of the aforementioned parameters. For example, although we did find the USA300 isolates to be highly hemolytic, strain IA2980 was an obvious exception. Both the USA100 and the USA600 lineage had isolates that were high or low in hemolysis. Intraclonal variation in biofilm formation was also observed, but it was specific to the USA300 and USA600 isolates in our collection. The USA100, USA200, and USA400 isolates consistently produced strong biofilms. Furthermore, blood survival variability was also observed among all clonal groups and was independent of the isolation site. *S. aureus* strains isolated from blood exhibited as much heterogeneity as the rest. We found no correlation between hemolytic potential and survival in the blood. For example, strains IA116 and IA209 (USA100) and strains PSLV and IA1012 (USA300) are some of the most highly hemolytic strains in our collection, and yet these strains do not exhibit a commensurate increase in blood survival. Conversely, strains MN8 and IA1101 (USA200), MW2 (USA400), IA88 (USA600), and Col are low in hemolytic activity, and yet they exhibit high blood survival. Whether biofilm formation can compensate for reduced ability to survive in blood, allowing hematogenous spread by USA100 strains, is currently unknown. It is also unknown whether increased ability to survive in blood compensates for reduced ability to form biofilms in USA600 strains. Which combination of traits contributes to or is required for specific pathologies is also undetermined. These are areas of interest currently being pursued by our laboratory.

It is evident that the mechanisms contributing to invasive diseases like infective endocarditis and lethal sepsis are multifactorial and not intrinsic to any one individual clonal group. High cytotoxicity is not an intrinsic property of USA300 strains alone, as many USA100 and USA600 isolates also have high cytotoxic activity. Biofilm formation, as tested *in vitro*, is not an intrinsic property of all *S. aureus* isolates, as many isolates belonging to the USA300 and USA600 lineages form poor biofilms. Finally, the production of high-level SAgs, such as TSST-1, SEB, and SEC, is also not intrinsic only to USA200 and USA400 isolates, as many USA600 isolates and some USA100 isolates also produced SEC. Therefore, it is important to look at multiple parameters when determining strain virulence and to be cautious about classifying strains with low cytotoxicity as avirulent. Strains with low cytotoxicity can have increased virulence due to the production of a high-level SAg (exemplified by *S. aureus* strains MN8 and MW2) or cause serious invasive disease due to their proficiency in key functions like blood survival and/or biofilm formation (exemplified by *S. aureus* strains IA1871 and Col). The infective endocarditis and sepsis studies further highlight the importance of expanding our knowledge of *S. aureus* pathogenesis to include isolates belonging to clonal groups associated with invasive disease and high mortality worldwide, namely, isolates from the USA100/CC5, 200/CC30, and 600/CC45 lineages (4, 5, 17–20). This understanding will make it possible to develop intervention strategies directed toward the most effective targets.

## MATERIALS AND METHODS

### Bacterial strains and growth conditions.

The *S. aureus* strains used in this study are listed in Table 2 and were used at low passage. Most of these *S. aureus* strains come from the Minnesota and Iowa collections. The strains were cultured in Todd-Hewitt (TH) broth (Becton, Dickinson, Sparks, MD) at 37°C.

### Superantigen and cytolysin gene screen.

Genomic DNA was extracted from overnight cultures using the DNeasy blood and tissue kit (Qiagen, Inc., Valencia, CA). PCR amplification of superantigen genes was carried out using *Taq* polymerase (Qiagen) or Phusion HF DNA polymerase (New England Biolabs, Ipswich, MA) with superantigen-specific primers (28, 47). Cytolysin genes were detected by colony lysis PCR using Phusion HF DNA polymerase. *hla* was detected using primer set hlaBamHIfor (5′ TTACGGATCCATGAAAACACGTATAGTCAGCTCAGTAAC 3′) and hlaXhoIrev (5′ TTACCTCGAGATTTGTCATTTCTTCTTTTTCCCAATCG 3′). *hlb* was detected with hlbscreenF (5′ CATCAACTGTTGCTGAAGATGGC 3′) and hlbscreenR (5′ CGGTTTCTCAGTAACAACTTCATTGAC 3′), a primer set that detects the gene regardless of phage integration. *pvl* (*luks*) was detected using primer set pvluksfor (5′ GACTATTAGCTGCAACATTGTC 3′) and pvlluksrev (5′ CCAGTTCACTTCATATTTAACTG 3′).

### Superantigen expression assays.

SEB, SEC, TSST-1, and SE*l*-X were detected using standard Western blotting techniques. Briefly, organisms were cultured in TH broth (Difco Laboratories, Detroit, MI) at 37°C with shaking overnight. The supernatants were concentrated by precipitating them with 4 volumes of 100% ethanol overnight at 4°C, centrifuging at 4,000 × *g* for 10 min, and resolubilizing in distilled water to 1/20 the original volume. Western immunoblotting with specific polyclonal rabbit antisera prepared against the designated SAgs was used to quantify the amounts of SAgs produced by each isolate. Purified SAgs were used to generate standard curves for quantification.

### Rabbit erythrocyte lysis assays.

Overnight cultures diluted to 1 × 10^9^ CFU/ml in phosphate-buffered saline (PBS) were spotted onto rabbit blood plates (RBP) in 5-µl volumes and incubated at 37°C with 5% CO_2_ for 18 to 24 h. The areas of hemolysis were calculated with ImageJ (48).

### Biofilm assays.

The well-established microtiter biofilm assay was used (49), with 3 × 10^5^ CFU/ml bacteria in tryptic soy broth (TSB) (Becton, Dickinson, Sparks, MD) supplemented with 2% glucose and 2% sodium chloride growing in plates coated with 5% human plasma (Innovative Research, Inc., Novi, MI). The plates were incubated overnight at 37°C with 5% CO_2_. The absorbance of solubilized crystal violet was measured at an optical density at 562 nm (OD_562_) (Tecan Systems, Inc., San Jose, CA). We performed one-way analysis of variance (ANOVA), the Holm-Sidak multiple-comparison test, and individual pairwise comparisons derived from *post hoc* tests. This resulted in roughly four statistically significant groups, as follows: (i) strains producing biomass mean values (OD_562_) of less than 0.5; (ii) strains producing biomass mean values equal to or higher than 0.5 but less than 1.0; (iii) strains producing biomass mean values equal to or higher than 1.0 but less than 1.5; and (iv) strains producing biomass mean values equal to or higher than 1.5. Each group was comprised of strains with biofilm formation that was not statistically different than that of the other strains within the group but was statistically different than strains in the groups with higher or lower biomass values.

### Blood survival assays.

Blood survival assays were performed as previously described (50). Bacteria were inoculated at 1 × 10^7^ to 5 × 10^7^ CFU/ml into 1 ml of freshly collected heparinized rabbit blood. Samples were incubated at 37°C with 360° rotation for a total of 120 min. Samples were collected at time points indicated above. Percent survival was calculated as the bacterial count recovered at a specific time point divided by the bacterial count at time zero.

### Rabbit model of infective endocarditis and sepsis.

The native valve infective endocarditis and sepsis models were performed as previously described (42, 51). Young adult (2 to 3 kg) New Zealand White rabbits, male and female were used. Briefly, a hard plastic catheter was inserted through the left carotid artery to reach the aortic valves, left in place for 2 h to induce valve damage, and removed. Bacteria were injected intravenously, seeding onto damaged valves and quickly forming vegetations (visible by necropsy in 24 h) that grew over the entire test period (4 days). All rabbit experiments were performed according to established guidelines and a protocol approved by the University of Iowa Institutional Animal Care and Use Committee (protocol 1312218).

### Statistical analyses.

Statistical significance in survival experiments was determined using the log-rank Mantel-Cox test (GraphPad Prism Software). Significance across means was carried out using one-way ANOVA and the Holm-Sidak multiple-comparison test (GraphPad Prism Software) and *t* test analyses derived from *post hoc* tests.
